# "The Hospital" Nursing Mirror

**Published:** 1901-08-03

**Authors:** 


Supplement to The Hospital, August 3, 1901.

### "The Hospital" Nursing Mirror.

Being the Nursing Section of "The Hospital."

[Contributions for this Section of "The Hospital" should be addressed to the Editor, "The Hospital" Nursing Mirror, 28 & 29 Southampton Street Strand, London, W.C.]

### Notes on News from the Nursing World.

### WAR NURSES AT MARLBOROUGH HOUSE.

Yet one more function of great interest to nurses took place at Marlborough House on Monday afternoon. After the King had presented several hundred soldiers who served in South Africa or during the Ashanti campaign with war medals, the military nurses and nursing sisters who have ministered to the sick and wounded received from his Majesty a similar token of honour. It may be added that though this was the last presentation of the season, those nurses who have been working in South Africa and have not so far received medals should communicate with the Army Medical Department, by whom, we are assured, their claims will be at once considered.

### THE WOMEN'S MEMORIAL TO QUEEN VICTORIA.

At a meeting of the general committee of the Women's Queen Victoria Memorial Fund in connection with the Queen's Nurses held last week, satisfactory progress regarding the fund was reported, and a full list of organisers of counties and boroughs of England and Wales was given. It was also announced that all the counties of Ireland were supplied with organisers under the Countess Cadogan's committee in Dublin, and that the Duchess of Buccleuch was completing her arrangements for the organisation of Scotland. A local committee has been formed in Berkshire; Lady Wantage has consented to act as president, and she has opened the subscription list with a contribution of £100.

### MISS FLORENCE NIGHTINGALE ON THE DISTRICT NURSE.

At the anniversary of the Guild of St. Barnabas for Nurses, which lately celebrated its twenty-fifth anniversary, the following pencilled greeting from Miss Florence Nightingale was read:—"May God bless and keep the nurses here in a body is the fervent prayer of Florence Nightingale. And He does bless them; He will bless them; He will make each feel her tender responsibility for the well-doing of all. The district nurse has more than all other nurses to seek wisdom and help from God alone—*to merge self in God*, and not only in God, but in each one of God's children whom she is nursing. She has not to be crucified like Christ, but she has, like Him, each in her humble way and seeking of God wisdom and love, to imitate His daily life of self-sacrifice."

### MATERNITY NURSING IN JAPAN.

A nurse in Tokio, who is occupied chiefly in nursing maternity cases, found it for some time a source of difficulty to dispose of the placenta or to get it disposed of. Not knowing the language, it was awkward for her to make the Japanese servants understand; and there are not many facilities for having it burnt on the little charcoal stoves, &c., which are used in that country. But quite lately she discovered that there is in Tokio a factory the business of which is to dispose of placentas. On receipt of a card or notification the company will send for it, and either burn or bury it, as desired, for a fixed price; in fact, there are three classes—first, second, and third—each having a different price. The first-class placentas are buried; the second and third class are burnt. There is another thing in connection with this branch of nursing as practised in Japan which is unusual, namely, not tying the cord on either the infant's or the mother's side, but simply cutting it after the pulsation has ceased. A custom very prevalent amongst the Japanese women is the wearing of a tight binder for many months before the confinement. This custom is very much condemned in the local papers as one of the chief reasons for the small stature of the people.

### PRIVATE NURSES AND THE PUBLIC.

The letter from the matron of a nurses' home at Preston which we print in another column is painful reading. We, of course, entirely agree with our correspondent that nurses "who get drunk on duty" disgrace both their uniform and their sex. But nurses who get drunk in any circumstances also disgrace their vocation, and the matron of a home who discovers that any of her nurses are addicted to drinking habits should dispense with their services forthwith. Our correspondent more than suggests that the public are to blame for not providing proper meals for the nurses engaged at serious cases, and that in Preston, at all events, they are often expected to do without food or sleep. Even the most utter want of consideration on the part of the friends of a patient is no excuse for a nurse fortifying herself with alcohol; but speaking generally, there is no doubt that if private nurses have their weaknesses, their employers are frequently in fault for failing to provide even absolute necessities. It is obviously desirable that black sheep should be weeded out of the ranks of nurses. But we question whether they are so numerous as our correspondent seems to think. All matrons should make most careful inquiries regarding the antecedents of their nurses before sending them out to private families, and we cannot but think that the experience of the writer of the letter alluded to is quite exceptional.

### COMPENSATION FOR LOSS OF OFFICE.

At the meeting of the Metropolitan Asylums Board on Saturday notice was given that the following recommendation of the Hospitals Committee would be moved at the next meeting on October 5: "That, subject to the approval of the Local Government Board, Miss H. P. Bunyan, a charge nurse at the Brook Hospital, be awarded a gratuity equal to the amount of one year's salary and emoluments, viz., £68 2s. 10d., for loss of office under circumstances which would be subsequently reported to the Board."

### THE HAMILTON ASSOCIATION.

The sixteenth annual report of the Hamilton Association for providing trained male nurses is very unsatisfactory. The committee have to make the discouraging announcement that during the year it has been necessary to still further draw on the capital to carry on the work, and to state that there has been an insufficient demand for the services of the nurses. The number of nurses on the roll the last day of June was 20, of whom 14 were employed, and it appears that 14 was also the average daily number employed in the past year. Nurses have been supplied to several of the leading London hospitals, and we are glad to see that the committee are not under the delusion that they would derive any benefit by lowering their standard of training. The competition of men who do not reach that standard has, they believe, increased their difficulties, but they intend to adhere to the policy which, as the pioneer in providing trained male nurses, the Hamilton Association has always observed. In the long run they will doubtless have their reward. There is no hope in the future for male nurses of inferior training, though they may seem for a time to command a measure of support.

### THE NURSES OF SALFORD WORKHOUSE INFIRMARY.

Much disappointment has been caused among the nurses of the Workhouse Infirmary at Salford by the recent decision of the Board of Guardians. It was quite anticipated that the scheme for the proposed home would be approved and forwarded to the Local Government Board for their sanction. It provides accommodation for 70 nurses and the servants at an estimated cost of £12,000, and the chairman of the Infirmary Committee assured the Guardians that it is urgently needed. But as a resolution in favour of proceeding with it was defeated, the matter is, at present, in abeyance. The only consolation for the nurses is that as the number of patients in the infirmary continues to increase, something will have to be done. The home is not only required for their comfort and convenience, but also because its erection would enable the guardians to add to the number of beds.

### A HOSPITAL GARDEN PARTY.

Last Thursday a remarkable party was held at the Norfolk and Norwich Hospital. Its principal object was, as a nurse at the hospital writes, to bring more prominently before the eyes of the public this institution which is doing such excellent work among the poorer classes. Upwards of 3,000 invitations were sent out, and there were 2,000 acceptances. The reception by the Mayor and Mrs. Dawson took place at 4 o'clock, and then everyone passed on to the wards, which were thrown open and beautifully decorated. All available space in the building was filled with pots of flowers and palms. Down the centre hall were large blocks of Norway ice, ornamented with smilax and various grasses, all helping to render the place cool and pleasant. In the grounds were three large tents for refreshments, with the hospital colours—red and white—floating above. The party was most admirably arranged and carried out from beginning to end. The Blue Hungarian Band played a most charming selection, which was evidently enjoyed as much by the patients and nurses as by the visitors.

### A NEW DEPARTURE AT BLACKBURN.

At the annual meeting of the Blackburn District Nursing Association, a very definite scheme for advancing its interests was decided upon, at the instance of Mr. J. S. Pollitt. Henceforth, each ward in the town will have its own lady treasurer, and she in turn will select her own assistant, so that, as Mr. Pollitt observed, "each ward becomes a complete unit, with a network of machinery for disseminating information and for adding names to the subscription list." By means of this organisation we do not doubt that the association will receive not only more support, but will also be kept in touch more fully with all its existing subscribers. It has now been in existence for five years, and the rate of progress has been satisfactory. But the managers of the association have wisely taken a step which should help to facilitate the development that cannot fail to be required.

### A HOME FOR CHATHAM.

The proposal to found a home for the nursing staff at Chatham as a memorial of Queen Victoria has been taken up very warmly, and a number of subscription have been promised. It has been suggested that the Town Council should vote £200 from the borough fund, and altogether there is every indication that the movement will be successful. The testimony of the Mayor is that the nursing institution is doing "a noble work among the poor of the town." In these circumstances it will surely be the pleasure, as it is the duty, of the well-to-do people of the town to provide the staff with a home.

### DISTRICT NURSING IN CHICHESTER.

According to the annual report just issued the District Nursing Branch of the Chichester Infirmary is being carried on with great advantage to the poor of the city. Last year 41 patients were nursed and 3,579 visits were made. Moreover, the lady visitors at the infirmary and their friends were able, through the agency of the district nurse, to contribute many medical comforts to the patients. The accounts of the branch are, of course, kept quite distinct from the general fund of the infirmary, no portion of which is applied to the purposes of district nursing. We are glad to learn that the adverse balance last year has been converted into one, though of a very small character, on the side of profit. Further pecuniary support is still needed in order to place the organisation upon a sound financial basis.

### GRANTHAM IN NEED.

The report of the Grantham Victoria Nursing Association, which was adopted at the annual meeting, is not satisfactory. The work of the nurses has been admirable; and the number of visits paid was 10,694 as against 2,085 two years ago. But while this shows how the demands for the services of the nurses has increased, and how much they are appreciated, we regret to observe that the subscriptions and donations only amounted to £127, or nearly £100 less than the expenditure of the Association. In these circumstances the committee have very properly suggested that the artisan population, who derive the principal benefit from its existence, should be invited to augment the funds. Judging by the experience of other towns, some of them not so prosperous as Grantham, the working men only require to have the matter brought systematically before them to ensure a reasonable response.

### DISTRICT NURSES' ENTERTAINMENT.

For some time past the nurses of the Hammersmith and Fulham District Association had been anxious that Thursday afternoon should be fine, for an entertainment was being arranged to take place in the garden of Carnforth Lodge. The garden is their special pride, each nurse tending her own plot; and the rows of sweet peas and hollyhocks were all ready for the occasion when, after a sunny morning, the storm broke, and the rain came down in torrents. To have the acting on the lawn was out of the question, but strips of Indian matting were laid down on the gravel path outside the French windows, and here the spectators who trusted that something would be done to avoid total disappointment, assembled at five o'clock, and the children of St. John's Band of Hope gave a musical play entitled "Cinderella and the Prince." Among the guests was a patient in a bath chair, who had not been out for years; and it is to be hoped that there was not a great deal of additional work in the district the following day. The proceeds amounted to something over £2. The refreshments, which it was announced could be procured during the interval at popular prices, were given away in consideration of the weather. One other item on the programme was: "A stall of vegetables and flowers grown on the premises—all fresh and cheap!"

### DEDICATION OF A MEMORIAL AT WALLINGFORD.

LAST Thursday evening a very interesting and impressive ceremony took place at Wallingford Cottage Hospital, when Canon Sir John L. Hoskyns dedicated the new bed which has been endowed by the relatives and friends of the late Miss Helen Mary Webb. A sum in excess of the £1,000 necessary for the memorial was readily forthcoming, and the whole of this amount has been devoted to the endowment. At the close of a suitable address Canon Hoskyns offered intercessions for the matron in charge, the patients and nurses, the managers and the donors. In the inscription on the Helen Mary Webb bed are these words:—"Endowed in thankful appreciation of her character and constant desire to minister to the wants of others, especially for the sick and suffering." As the matron of the Wallingford Hospital, in sending on the report, says, "It may be the means of suggesting a way of helping other cottage hospitals."

### TRAINING PROBATIONERS AT HULL WORKHOUSE INFIRMARY.

THE Hull Board of Guardians some time ago determined to try and solve the difficulty of obtaining nurses by offering to train probationers at the workhouse infirmary on nominal terms. It seems, however, that the experiment was undertaken without proper consideration. At the last examination three out of the five candidates submitted for examination failed, and the examiner, in his report, stated the reason to be that they had no means of obtaining a sufficient knowledge of their work in all its branches. The principal points in which he found the candidates wanting were surgical nursing, sick room cookery, knowledge of nursing apparatus and instruments, and generally an absence of the power of observation and inability to deduce or to understand the connection between cause and effect. To overcome these deficiencies he has recommended the reception of surgical cases at the workhouse, cookery lessons, demonstrations of apparatus, and periodical examinations. In consequence of this report the Staff Committee have since gone into the matter thoroughly, and at their instance a series of suggestions have just been adopted by the Board which, it is hoped, will put the training of the probationers on a proper basis. It is, of course, far better not to attempt to train than to do it in a manner which cannot fail to be unsatisfactory all round.

### COMPLIMENTS TO A MATRON AND NURSING STAFF.

AT the annual meeting of the Retford Hospital and Dispensary, general satisfaction was expressed at the manner in which Miss Babcock, the matron, formerly matron at the Luton Bute Hospital, has acquitted herself. In their report the committee stated that "under the judicious management of the matron, Miss Babcock, the efficiency of the hospital and the domestic staff has been fully maintained, notwithstanding exceptional difficulties caused by the deficiency of fully qualified nurses, and the committee's recognition of the services to the hospital of the matron, out-nurse, and probationers is hereby recorded.' The Chairman, in the course of his speech, also observed that they were thoroughly satisfied with the way in which the matron had carried out her duties; and the following extract from the report of the medical officer was read:— "During nearly the whole of the present year Miss Babcock has been the matron, and we cannot speak too highly of her skill, or of the care and interest she takes in the patients and the welfare of the hospital. The other members of the nursing staff we find always obliging, kind to the patients, and taking an intelligent interest in their work." Compliments of this kind, though frequently well earned, are not, perhaps, so often forthcoming as they might be. Neither matrons nor nurses are likely to do their work any the less efficiently because they occasionally receive a few judicious and well-timed words of recognition.

### SAD DEATH OF A NURSE.

THE death of a nurse attached to the staff of Chippenham Workhouse Infirmary under very distressing circumstances is reported. The deceased, Miss Florence Stanley, who was engaged to be married, was spending a portion of her holiday at Calne with her uncle, in company with her fiancé. He left on the Monday morning, and she went with him to the station. The report states that they parted on affectionate terms, and that Miss Stanley returned to her uncle's house, but refused to have any breakfast. She then wrote a letter addressed to her fiancé and left the house again. She was seen to go along the canal bank, and subsequently her body was found in the canal. At the inquest the jury returned a verdict of "asphyxia from drowning during a fit of temporary insanity," no motive being suggested for the suicide of the unfortunate nurse.

### "QUEEN'S" NURSES AT HUDDERSFIELD.

THE work of the Huddersfield and District Victoria Sick Poor Nurses' Association, which is affiliated to the Jubilee Institute for Nurses, continues to develop. It was stated at the annual meeting that during the year there were 614 applications for the nurses' services, as against 496 in the previous year; that 12,364 visits were paid, as against 10,945; and that the operations attended by the nurses were 137, as against 68. The chairman, in the course of his speech, laid stress on the importance of the last increase, and said that in many cases, but for the assistance of their nurses, the patients would have to be removed to the Infirmary, or the operations would have to be performed under circumstances very much less satisfactory to the doctors who undertook them. The nurses are now established in their comfortable new home.

### LOWESTOFT PROVIDENT NURSES' ASSOCIATION.

A VERY successful garden fête and fancy bazaar has been held in the grounds of Gunton Old Hall, on behalf of the Lowestoft Provident Nurses' Association. Mrs. Lucas, wife of Colonel Lucas, the member for the division, performed the opening ceremony, and subsequently the latter spoke in the highest terms of the work done by the nurses. The Association owes its existence to the initiative of Lady Cunliffe Owen, who not only worked hard to advance its welfare, but also raised the sum of £500 among her friends as an endowment. Owing to many calls the committee were obliged to use £100 of this money, and the primary object of the bazaar was to enable them to replace it, and keep the endowment, as it should be kept, intact.

### Lectures to Nurses on Medicine and Medical Nursing.

By NORMAN DALTON, M.D. London, F.R.C.P., Physician to King's College Hospital.

LECTURE XVI.—DISEASES OF THE ORGANS OF RESPIRATION—(*Continued from page* 208).

THE EXPECTORATION—*Pneumonia*—*Bronchitis*.

THE next point to consider about the diseases of the respiratory organs is the expectoration or sputum. This should, of course, be always kept for the physician to examine, and if the disease be an infectious one, such as diphtheria, the spittoon should contain about an ounce of 1 in 20 carbolic acid, and should be frequently emptied. But if the sputum be wanted for the purpose of cultivating the bacteria which it may contain, it must not be touched with any antiseptic, but should be placed in a clean bottle or test tube, the orifice of which is then plugged with cotton wool; or corked and sealed up if it is to be sent by post. The bottle should be first boiled for half an hour (if time be no object) and kept plugged with cotton wool until it is cool. If a small but wide mouthed bottle can be obtained, the patient should, if possible, expectorate directly into it. Any membrane or fleshy-looking material which may be expectorated, and which may be wanted for microscopical examination, should be put into methylated spirit mixed with an equal quantity of water. If it were allowed to become dry it would be quite spoilt.

In acute inflammation of the lungs (croupous pneumonia) the characteristic sputum is of an uniform reddish colour and is called "rusty sputum." It is very tough, is expectorated in pellets and clings to the bottom of the spittoon. The uniform colour is due to the intimate mixture of red blood corpuscles with the rest of the sputum. There may be streaks of pure blood present, but that would not be typical. An attack of pneumonia begins very suddenly with a severe rigor, often accompanied by vomiting. There is severe dyspnœa, the breathing being often 60 to the minute, while the pulse is 120. It will thus be noticed that the relation of the pulse to the respirations is greatly altered, being 2 to 1, instead of 4 to 1 as in health. The relation between the pulse and the number of respirations is always an important thing for nurses to observe. In many diseases, the fevers for instance, both are proportionately increased, *i.e.*, if the pulse be 100, the respirations are from 20 to 25. In some affections, such as Grave's disease, the first stage of tubercular meningitis, etc., the pulse is very rapid while the number of respirations is only moderately increased; while in pneumonia, as we have seen, the reverse is the case. The distress of the patient at the beginning of pneumonia is extreme, partly from the dyspnœa and partly from a severe pain in the affected side, usually situated in the lower part of the axilla and much increased on coughing; but nurses may comfort their patients by telling them that both the pain and the sense of suffocation will become less as the disease progresses. At the close of a fatal case, however, the extreme dyspnœa usually returns. From the outset, the temperature in pneumonia runs high. It may be 104° or 105° on the first day. The skin is burning hot, the face flushed, the pulse full and bounding, and headache is often very severe. In many cases a crop of red pimples appears on the lip or cheek, and after about 24 hours each pimple becomes filled with a clear yellow fluid. This eruption is called " herpes," and it is the same eruption as the one called "shingles," which occurs round the trunk. A case of pneumonia lying in a ward is often detected from a distance by the herpes on the face The characteristic rusty sputum may be expectorated at any time after the first day. As a rule, a case of pneumonia lasts without much variation in the symptoms from 7 to 10 days, and then terminates by "crisis," *i.e.*, the temperature comes down in the course of a few hours from, perhaps, 104° to normal or to below normal. The patient may sweat profusely during this period, but feels immensely better, although by examination we find that the changes in the lung take some days more to clear up. Severe cases of pneumonia are characterised by a very high temperature, and great dyspnœa. In persons who have been intemperate, delirium is very likely to occur. The delirium often, takes the form of delirium tremens, (*i.e.*, the patient is noisy and sees imaginary insects, etc.), and cases of pneumonia have been actually mistaken for cases of delirium tremens, which is a very fatal error, for opium and other sedatives, which are so useful in the latter, are most injurious in the former. The mistake is the more likely to occur because in intemperate people and also in very old people, the temperature may not be very high, there may be no cough to speak of and the dyspnœa may not be marked.

The strength of a patient with pneumonia must be preserved by the measures mentioned in the lecture on the Nursing of Disease of the Heart. The nurse should be on the look-out for two symptoms, both of which are extremely unfavourable. One is the occurrence of sputum, which is not rust-coloured, but dirty brownish-red, like "prune juice;" the other is an irregular temperature. In favourable cases the temperature remains almost at the same height every day and night until the "crisis" occurs. If, therefore, there are irregular falls and rises, and, in particular, if the temperature rises to a considerable height after the crisis, the outlook is very unfavourable. The pulse is not a good guide to prognosis, and it may remain full and strong to within a few hours of death.

There are certain cases of acute bronchitis which begin in the small bronchial tubes, and are usually associated with patches of pneumonia scattered through the lungs. These are called cases of broncho-pneumonia, because both bronchitis and pneumonia are present. They occur most frequently in children, and in adults who are suffering from influenza, and they are always very serious. On the other hand, ordinary cases of acute bronchitis usually begin in the big bronchial tubes at the bifurcation of the trachea, for which reason the patient suffers, at the commencement of the attack, with a severe pain behind the middle of the sternum. There is only slight fever, but the cough is very frequent and greatly increases the above-mentioned pain. For the first few hours no true sputum is brought up, only a little thin frothy fluid which does not come from the affected part of the tubes. The first true sputum is thick, pellety, and greyish-yellow in colour; and the expectoration of it gives the patient relief. Later on the sputum becomes thinner, frothy, and more yellow; and in bad cases the quantity of secretion in the tubes may be so abundant and the difficulty in coughing it up may be so great as to cause death from suffocation.

In the cases of chronic bronchitis and emphysema which are so commonly met with in elderly people, the sputum is comparatively thin and abundant. If it be exceptionally thin and very abundant, the condition is called "bronchorrhea," which means a running from the bronchial tubes. Coalminers and other persons whose work entails the continual breathing of coal dust often suffer from chronic bronchitis, and their expectoration may contain so much of this dust as to be quite black.

The sufferings of patients who are ill with severe bronchitis, whether acute or chronic, resemble closely those of patients with uncompensated mitral disease. There is the same continuous shortness of breath, with orthopnea and inability to sleep, the respiratory muscles ache from overwork, and the muscles of the limbs from the cramped posture. There is not, however, as a rule, any dropsy, either of the feet or the trunk. They must be nursed in the same way as cases of mitral disease, every effort being made to preserve the strength, so that all the patients' power may be devoted to the heart and the respiratory muscles. It is important to note that a poultice weighs a considerable amount, and that at each breath the muscles have to expand the chest plus lifting the weight of the poultice on it. So a poultice adds to the amount of work to be done and many physicians stop poulticing when the patient shows any sign of weakness. If they are ordered, the nurse must be careful to make them as light as possible.

### Sick Quarters for Sick Nurses.

By A Correspondent.

It is somewhat curious, despite the progress and improvements which the last fifteen years have added to our hospitals, that some of our largest training schools remain without a nurses' sick quarters.

### "Warding" Sick Nurses.

There are many and various ways of providing for the sick among the staff. The barbarous practice of "warding" sick nurses is still in vogue—notably in one of the largest and richest among the London Hospitals. In other instances the nurses' home serves as an impromptu sick quarters for nurse invalids. Nurses, too, suffering from a slight illness lasting a few days only sometimes continue to share a room with another nurse, a state of things which is neither pleasant nor hygienic for either. The first essential of illness is quietude; and no nurses' home can be said to include this elementary principle of nursing science. The constant tramping up and down of the bare-boarded stairs and passages, the early morning baths, the exuberant spirits of nurses going off for half days, all combine to make the nurses' home a very uncomfortable sick quarters.

### The American Plan.

It has often been urged that every private house should contain a little nook specially planned and designed for the use of possible patients of the household. Without going quite so far as to advocate this as a necessity to domestic life, I think there cannot be two opinions as to the need of a properly organised sick quarters attached to every hospital. The first points of admiration felt by an English visitor to American hospitals is brought out by the admirable little nurses' infirmary belonging to most of their training schools. In this matter, and in some others, we are quite behind our Transatlantic cousins.

### An Object Lesson.

A recent influenza epidemic in bitterly cold spring weather furnishes a striking object lesson of the discomforts and dangers suffered by sick nurses for whom no adequate provision is made. Some thirty of the staff of a large London training school were "down." Some of the worst and the lung cases were "warded," to their infinite discomfort and to the obvious detriment of the other patients. In itself the fact of being "warded" is not a pleasant circumstance. The noise, the publicity, and the necessary ward routine are irksome hardships to the nurse, coming as she does from a class very different from that of the average patient to whom custom has commonly made such things a matter of indifference. Of these thirty influenza nurses some twenty-five were distributed in the Nurses' Home. The night nurses attacked kept to their own quarters—the day staff remained in their normal cubicles. Thus scattered, it is obvious that the nursing was conducted on very unsatisfactory lines. As one of the nurses told off to succour her distressed sisters put it, "Some were in Number Two, others in Fifty-nine, so that my time was practically spent on those cold, draughty stairs, and 'nursing' in any proper sense of the word was impossible." Keeping up the fires in so many individual bedrooms was a physical impossibility. Many of the servants and wardmaids were affected by the epidemic so that both nursing and domestic staffs were short-handed. Consequently, the scattered sick nurses had a very poor time—many of them without a glimmer of fire throughout their illness. The food carried to such distances was cold and spoiled before it reached the invalids. Indeed from the nursing standard a mild chaos reigned supreme. And this in one of our most important hospitals where the nursing arrangements are boasted of as being up to date and complete.

### What Might Have Been.

A properly equipped nurses' sick quarters with an attached diet kitchen would have minimised the hardships of the situation. The worst cases would have been carefully nursed in their own sanatorium and thus saved the wretchedness of being warded. The cases came in crops, so that a sick quarters capable of holding 15 patients would have entirely met the requirements even of a serious influenza epidemic. A nursing sick section should, of course, be taken to include the sick among the domestic staff, and such a provision should be regarded as a *sine quâ non* of a hospital of modern times. Usually the invalided among the servants and wardmaids, unless they are ill enough to be relegated to the wards, have a very sorry time owing to the unsuitability of the servants' dormitories to the needs of the sick—but that is another story. At the present time I am dealing with the question of the absolute pressing need of a sick corner in every hospital furnished, garnished, and swept, standing ready at all times for any members of the staff who fall sick by the way.

### Nurses Worth Preserving.

Of course, such a scheme would cost money. But it is not a logical hospital programme to spend largely and generously on the patients in the wards, and to economise in the matter of the accommodation and care of its sick officers. Nurses are made of such good material as to be well worth preserving. And, after all, the hospital on whose staff they are has a moral liability towards them, and should give them every attention when disabled in the discharge of their duty.

### "The Hospital" Convalescent Fund.

The Hon. Sec. acknowledges with many thanks the receipt of 10s. from Miss E. M. Williams.

### To Nurses.

We invite contributions from any of our readers, and shall be glad to pay for "Notes on News from the Nursing World," or for articles describing nursing experiences, or dealing with any nursing question from an original point of view. The minimum payment for contributions is 5s., but we welcome interesting contributions of a column, or a page, in length. It may be added that notices of entertainments, presentations, and deaths are not paid for, but, of course, we are always glad to receive them. All rejected manuscripts are returned in due course, and all payments for manuscripts used are made as early as possible after the beginning of each quarter.

### Beyond the Seas.

### NURSING AT WINNIPEG GENERAL HOSPITAL.

I HAVE told you, writes a Graduate of 1899, in previous numbers of my first experiences as a probationer-nurse in Winnipeg General Hospital. On returning to hospital after my month's holiday I expected to find the usual ward work almost as hard as I had found it when I entered, so that it was with much pleasure that I heard my fate was to be the private-ward flat, as second nurse.

### The Private-Ward Flat.

The flat consisted of seven private wards, a private operating-room, bath-room, sun-gallery and kitchen. As luck would have it the work at that time was particularly easy, for though all the wards were occupied as a rule, the cases were chiefly surgical, and did remarkably well, so that apart from the ordinary routine work there was little to be done. The head nurse was the most lenient it has ever been my pleasure to work with, for she always insisted on taking the hardest and most disagreeable work herself, giving me the easiest and most interesting cases to attend to. I cannot say that I felt exactly happy under this *régime*, as I had an uncomfortable feeling that I was not doing my fair share of the work, but if I ever ventured to remonstrate she assumed a decided air of seniority just for the occasion—for I never saw it at any other time—which impressed me immensely, and I retired humbly to the making of superfine egg nogs, the arrangement of the ward linen into intricate and elaborate patterns, and other tasks of a like easy and eminently satisfactory nature.

### Beautifying the Operating-Room.

The operating-room we only attended during dressings from our own flat; when an operation took place the nurses from the theatre downstairs took charge. The room, however, and its belongings were ours to keep clean and make as beautiful as we knew how and cared to, and any spare time that we had was generally devoted to that task. By some means we persuaded the engineer to give us a quantity of white enamel and gold paint, and with that we went ahead to make everything shine; the radiators claimed our attention first, and here the gold paint proved useful. I remember the morning that I spent on my knees painting them. The head nurse promised to attend to the whole flat so that I could have an entire morning's work with my paint brush. The coils were hot and the gold paint smelt abominably as it dried on the pipes, but though I almost choked with the vapour rising from the gold leaf, I went valiantly on with my work determined to get it done in one morning. Then, the solution bottles; what hours we spent cleaning and polishing them with wonderful mixtures of our own invention, in which oxalic acid and washing soda figured largely, but the exact recipe of which we kept a dead secret only to be made up and used on our own flat. When they were finished and filled with clear carbolic or boracic solution we thought they could only be surpassed by our equally beautiful electric-light globes, or the polish of the dull green glass of the operating table. Our regret was great when some time during the day an inconsiderate doctor came along and would insist on doing a dressing in that exquisite temple dedicated to cleanliness and smelling delicately of methylated spirits and turpentine.

### Christmas in the Hospital.

Christmas drew near and great preparations as usual were made for it. Every head nurse and the juniors under her in the ward vied with each other in trying to make their part of the hospital the most attractive on Christmas Day. The private-ward flat hardly entered into this competition, for, except making lamp-shades for every room and arranging the lovely flowers which always arrived in abundance about Christmas time, the hallway and passage were the only places we could really decorate. The public wards, however, always looked charming, the usual mottoes and evergreens were supplemented with garlands and sprays of paper flowers and leaves, which some of the nurses were most clever at making; not those terrible paper "roses," but artistic trailers of convolvuli, vegetable marrow plant, purple iris, and chrysanthemum. On Christmas morning a choir from one of the city churches came at 10 o'clock to sing hymns and carols, to the delight of the patients, and at 1 o'clock dinner was served in each ward, the tables looking lovely covered with roses and other exquisite flowers. In the afternoon a Christmas tree for the children was an attraction, with charming dolls, books, and toys, to delight the hearts of the tiny ones. The nurses had no meal at the usual hour of 12 o'clock—for we were far too busy and excited to think of eating—but in the evening, the superintendents, house-surgeons, and nurses dined together in the nurses' dining-room. Just for that occasion seniority was forgotten, probationers dared to laugh out loud, staff nurses talked to mere juniors, the house-surgeons were positively frivolous, and the superintendents beamed upon us all.

### A Hospital Alphabet.

I remember that particular Christmas because two of the nurses took upon themselves to write a hospital alphabet in verse, which was read aloud after dinner and greeted with laughter on all sides as nearly every verse described one of the hospital staff. The only two verses which were not personal, and which I recollect (of course, I can remember all the more personal ones), were:—

> O for Oxalic, speak under your breath,
> Don't use on the bath, for the penalty's death.
>
> and
>
> S for Sapolio, scrub, and be meek,
> Remember probationer, just one cake a week.

Our hospital was managed on strictly economical principles, and the "Sapolio" verse appealed to us all, as the one cake allowed each ward during the week was always a sore point, and any strategy was considered fair to secure a second cake whether we wanted it or not. The lady superintendent was asked to read the verses aloud, as a great favour, by the authors, but no one else knew who wrote them, and the authorship remained unknown for some long time after.

### The Isolated Hospital Again.

At the beginning of January I was transferred to the Isolated Hospital again for duty in the scarlet fever ward. The five patients who awaited me were four children—all convalescent—and a nurse who had contracted the disease while on duty in the ward. Several new cases came in during the month, and at the end of that time I went downstairs to night duty. I felt rather nervous at this change at first, for it was the first time I had been the senior nurse on duty at night, and I had now to assume some of the duties of night supervisor. All the nurses in the infectious wards were my juniors, and though I never entered their sections except in emergencies, when they were in doubt I was the one to be appealed to. I had three wards to attend to; many of the patients were chronic cases and required little attention at night. However, I was often exceedingly busy, so much so that before I left another nurse came on to assist me.

*(To be continued.)*

### Army Nurses and the South African Medal.

### PRESENTATION BY THE KING.

ON Monday afternoon upwards of a hundred nurses received the South African Medal from the King at Marlborough House. The day was beautifully fine, and the crowd began to gather very early in the afternoon. There was some uncertainty as to what there was to see; and many, unaware that he had already returned from Down Place, Berkshire, thought that his Majesty would drive past St. James's Palace, on his motor-car, after his week-end visit to Lord and Lady Alington. Standing well in front of the crowd lining the pavement was a small family of ragamuffins, quite *au fait* as to the day's proceedings, and only a little hazy as to personalities. Several false "kings" were announced in shrill London voices, and "Bobs" was spotted in hansoms under various guises. Once a very young officer with his arm in a sling did duty for the hero. The first nurse—a fair-haired lady in spectacles—drove up at about ten minutes to two, and after that time the others arrived quickly, in twos and fours, nearly all in hansoms or four-wheelers, and a few in carriages with friends. By-and-by, as officers and nurses came more rapidly, the crowd increased. It was a picturesque sight, for the cabs deposited their fares under the archway at the further side of the Friary Court of St. James's Palace, where they stood in groups, many accompanied by friends.

### ARRIVAL OF THE NURSES.

Meanwhile, a detachment of Guards marched out, reminding one of a youthful toy in which a double row of scarlet-coated men could be drawn out from under a cardboard arch on a strip of paper, so like "little tin soldiers" did they look, in their regular marching order. Having halted, they saluted Major-General Sir Henry Trotter, who was in charge of the arrangements. As the minutes went by more and more officers arrived, and more and more nurses—Army Superintending Sisters, in grey alpaca uniforms, Army Sisters in dark blue with circular scarlet capes, A.N.S. Reserve Sisters in dark blue cloaks with scarlet-lined hoods, and finally, two nurses, who were afterwards ascertained to be Colonials. Next came a detachment of Scottish Rifles; then some officers in hansoms, and as by this time the crowd had blocked the entrance to the courtyard, they were put down under the archway facing the road, and left to make their way into the enclosed square. Among the arrivals attracting the greatest notice were the swarthy Central Africans, marching in excellent time, under two native officers in large khaki-coloured turbans. "Loyal fellows!" said a lady in the crowd. "We're proud of you." Then, to the ragamuffins, "Why don't you cheer them, boys?" But the boys evidently did not see the force of cheering by proxy, and, besides, they were too busy watching the cabs putting down their fares. They were, moreover, a little puzzled at the arrival of so many blue-cloaked ladies among the scarlet and khaki. At length, however, light dawned, and the eldest boy, taking from his lips a dreadful little cigarette rolled in newspaper, spoke as follows: "Don't you understand? The soldiers has been wounded, and the nurses has been lookin' awfter them!" When the various contingents had been got into line for marching across to Marlborough House, and the crowd had fallen back to make way, the procession started. Very quickly they went by, the nurses keeping well in time, and in a few moments the entire five hundred "soldiers and military nurses" disappeared inside the grounds of Marlborough House to the accompaniment of cheers from the crowd outside. This was at twenty-five minutes to four. Inside the grounds the front line was within a few feet of the gravel walk fronting the main entrance to the Royal residence, and the remainder, in alternate rows, stood on the lawn, which has become historic ground to members of the nursing profession during the last few weeks. The Royal Standard was flying from the flagstaff on the roof, and the band of the Grenadier Guards was posted on the lawn. Lord Roberts was present, in undress uniform, and with him several officers.

### THE CEREMONY.

Presently the King appeared, wearing the uniform of a field-marshal. With his Majesty was the Queen, leading Prince Edward of Cornwall and York by the hand, and behind came Princess Victoria with the other two children of the Duke and Duchess of Cornwall and York. As their Majesties appeared the band played the National Anthem, and the King, the officers about him, and little Prince Edward stood at the salute. The Queen and the Princess were both in deep mourning, and the children in white—the young Princes being in sailor costume. The King took up a position beneath an awning, Queen Alexandra stood at his right hand, and on their Majesties' right and left respectively were Earl Roberts and Major-General Trotter. At a signal from the Commander-in-Chief the officers in the front line began to file past, each one saluting the King and Queen on passing, responding to the call of his name by General Trotter, and receiving his medal at the King's hands. The Queen took no part in the ceremony beyond saying an occasional kind word to one of the recipients and graciously acknowledging the salutations of all. Her Majesty regarded with marked sympathy the wounded Colonials, and she and the King were much interested in the nurses. The black troops, too, received marked attention. Among the nurses were two born in the Colonies, and by their Majesties' special request these were brought up separately at the close of the distribution and were honoured by a long chat with the King and Queen, followed by a hearty handshake from the Commander-in-Chief. Sister Grantham had the honour of presenting a bouquet to the Queen. When the last medal had been handed to its recipient and the parade re-formed, the band moved forward to a position immediately in front of the main entrance to Marlborough House, and played the whole body of troops and nurses past once more. At five minutes past four, to the strains of the "National Anthem," the crowd outside had the satisfaction of seeing soldiers and nurses re-appear. They marched quickly across the road, and in a few moments left the court in groups, some wearing the medal, others carrying it in a little cardboard box.

### THE MEDALS.

All the medals awarded to nurses were for service in South Africa. They have on one side the head of Queen Victoria, and on the other a design representing Peace calling on the multitude, while in the distance are the masts of ships. The medal is worn with a ribbon of blue and red and khaki stripes. Names of nurses who have been in South Africa as Army nurses are still being collected by the Army Medical Department, and in time all those who apply for them will receive this token of the country's appreciation of services given in time of war. Several nurses travelled from the North in order not to miss the ceremony, and returned immediately afterwards.

### The British Congress on Tuberculosis from a Nursing Point of View.

BY A MATRON WHO WAS PRESENT.

So many points of interest to nurses were discussed at the various meetings, both general and sectional, of the British Congress on Tuberculosis that it is difficult to decide exactly upon which to touch without leaving out of consideration important questions. The surprising statements of Professor Koch and the caution with which they were received by other eminent scientists, however, calls for attention. The great question as to the infection of milk from the udder of a tuberculous cow is of the utmost importance not only to nurses working in hospitals and sanatoria where, at any rate, the authorities endeavour to see that the milk supplied to the institution comes from a trustworthy source, but in a far greater degree to the district nurse whose patients depend upon the small retail dealer, and whose varying demands cause them to patronise wandering milk-carts. It is much to be feared that lazy and indolent mothers who have been bullied by the district nurse into boiling all milk before giving it to infants and delicate children will neglect this simple precaution. We all know, unfortunately, how difficult it is to impress the ignorant with the necessity of carefulness in this respect. Hitherto laziness and want of time in cases where the mother herself goes out to work have been the chief difficulties, and one expects that another factor will now be added in that interested milk (and meat) sellers will call the attention of their customers to Dr. Koch's pronouncement—"I should estimate the extent of infection by the milk and flesh of tubercular cattle and the butter made from their milk as hardly greater than that of hereditary transmission, and I therefore do not deem it advisable to take any measures against it"—and so encourage them in their neglect, the ventilation of the matter in the public press lending them considerable aid.

### The Reopening of the Sterilisation Question.

To the housekeepers of institutions, again, the reopening of the sterilisation question (without arriving at a definite conclusion) is somewhat vexatious. In many hospitals and sanatoria, especially the small and poverty-stricken institutions, where the kitchen staff is often barely adequate to meet the demands made upon it, the additional work of sterilising all milk has frequently been a stumbling-block to the cook, who regards it as a fad and unnecessary, and it seems a pity that, just when institution servants have been educated into accepting it as of vital importance, doubts, at present far removed from verification, should have been discussed and freely circulated.

### Cleanliness and Sputum.

Dr. Koch also drew public attention to another point of great interest to the district and parish nurse: "The patient is left without the nursing he needs because the able-bodied members of the family must go to their work. How can the necessary cleanliness be secured under such circumstances? How is such a helpless patient to remove his sputum so that it may do no harm?" The answer to these questions is in England to be found in our magnificent organisations of Jubilee and other district or parish nurses. To these belongs the duty of educating the poor consumptive at home, and on their shoulders rests an enormous responsibility in this respect. All such workers should be supplied with leaflets for distribution to phthisical patients for their guidance and the safety of those around them, such as are published by the National Society for Prevention of Tuberculosis, the National Sanatorium, Bournemouth, and various other such institutions. At one of the sectional meetings the question as to the proper disposal of sputum was thoroughly discussed, and it was interesting to observe that almost all authorities agreed that cremation was the perfect method in contradistinction to washing it away into the drains, the method not only adopted at present in nearly all our chest hospitals, but even at Nordrach, where "each bedroom is fitted with a pair of lavatory basins—one for washing and the other for spitting." The National Society for the Prevention of Tuberculosis also sanction the deposit of expectoration into the drains as an alternative to cremation.

### The Pocket Spittoon.

Professor von Schrötter, of Vienna, handed round for inspection among the audience various kinds of spittoons made from peat, which could be burnt with their contents. For use in private nursing these would be invaluable, but in charitable institutions the expense of daily providing the inmates with new spittoons would render their use out of the question. The Professor also submitted to inspection a pocket spittoon flask, which appeared to be a very great improvement on that invented by Dittweiler. The latter, although serving its purpose, is heavy and cumbersome, and a great objection in the eyes of women patients with their small pockets. The improved flask has a glass collar instead of the heavy metal one in the original pattern, otherwise the two are almost precisely the same, with the exception of the catch closing the lid, which in the new flask is of the hinge instead of the spring variety—by no means an improvement in my opinion. The price of the improved flask—also a question of importance—is 1s. against 3s. 6d. for Dittweiler's patent. It is most desirable that some such flask as this should be provided by district and parish nursing organisations to those consumptives unable to pay for them. The money so expended would be of immense service in helping to minimise the risk of infection to the community at large

### Promiscuous Spitting.

It is a little surprising that although the enormous danger arising from promiscuous spitting was very fully dealt with, and even legislative measures suggested to suppress it, no mention was made of the enormous influence that might be brought to bear upon those who in a few years will probably be great offenders as young smoking men among the labouring class by the machinery of the Board and National schools. Habits once formed are not easily eradicated. Lads who have outgrown the control of their schoolmasters cannot be coerced; but surely whilst young they could be taught the dangers of such a filthy habit and severely punished if caught practising it.

### The Disinfection of the Houses.

Closely connected with the disposal of sputa is the question of disinfection of houses, etc. Dr. Coates, of Manchester, having proved the infective properties of dust in houses where consumptive patients are living, and also the fact that its virulence can be long retained, recommended as thoroughly efficacious a solution of chlorinated lime, strength $1\frac{1}{2}$ oz. to the gallon, to be used as follows:—"The wall-paper to be thoroughly saturated with this solution, applied with a soft brush, and is then stripped from the walls. The bare walls, ceiling, and the floor are washed over several times with the solution, and any articles of furniture which will admit of such treatment are similarly washed over." On the other hand if great precautions have been taken as to the disposal of sputa Dr. Coates considers it sufficient to rub the wall-paper with crumb of bread or kneaded dough, whilst the floors, painted walls, and woodwork may be washed with soap and water and the ceilings lime-washed. In any case, however, the bedding, articles of clothing, etc., should be "either disinfected by steam or washed with boiling water."

### Methods of Ventilation.

Space forbids me to do more than just allude to the discussions of "the methods of ventilation and standards of pure air to be aimed at." It is interesting, from a nurse's point of view, to learn that "to warm the patient or the room itself in moderation is permissible; but the air cannot be too cold in this country for the good of the patient," and that both Dr. Walthers, of Nordrach, and Dr. R. Walters consider a good current of air through the rooms inhabited by consumptives desirable.

---

[We would point out that there are other diseases besides tuberculosis, and that, however right Professor Koch may turn out to be in regard to this disease, the question of the desirability of boiling milk is hardly touched by his conclusions.—Ed. HOSPITAL.]

### Lectures to Head Sisters.

By E. MARGARET FOX, Matron of Tottenham Hospital.

### LECTURE II.—ON WARD MANAGEMENT.

THE fact of a ward being well managed is so patent to the most careless observer, that no one can help knowing when such is the case. Every nurse, on being appointed ward sister, wishes to manage it well, and has her own ideas on the subject; but it is not everyone who realises that attention to details and a fair division of labour is at the root of all good management, and that to be a good ward sister you need to practise some of the most difficult and most unpopular virtues, and that, not once, but all the time. Patience, for instance, we find very hard to practise constantly, and the excellent business attribute of punctuality we shall all agree is extremely difficult to translate into action the first thing in the morning! But if you aim at becoming good ward managers, these attributes must be yours, together with kindness, firmness, truthfulness, thoroughness, order, and method, united with capability and a gift for teaching.

But these good qualities and habits are not acquired in a day, and even when acquired, need a good deal of keeping up to the mark. You have to supervise several people and many things to manage a ward well.

The visiting staff, the residents, your probationers and patients, their visitors, and various members of committees, all expect a due amount of consideration from you; and with regard to your ward, the furniture, linen, beds, clothes, food, medicines, and dressings, of course, come under your jurisdiction.

You are responsible for the cleanliness and order of every corner of your ward. No nook or cranny must escape your watchful eye, and no "glory-hole" should be allowed anywhere. You must see that the sweeping of walls and floor, the dusting, cleaning, washing, bedmaking, and feeding are all properly and regularly done by those whose work it is. If the buttons are continually missing from shirts and pillow-cases, and a spare bit of soap and flannel is never forthcoming for a new admission, if the patients are never washed at the right time, and the ward never in order by 8 P.M., if dressings are about all day long, and the ward kitchen and lavatory always in a muddle, then the conclusion one naturally draws is that the ward is not being well managed.

I have seen wards in which a certain picture would hang crooked for days, in which some of the blinds were permanently rolled up and fastened with safety pins, the cupboards always untidy, the patients' hair never brushed neatly, the beds invariably awry, the chairs uneven, and the meals not properly done with till the last thing at night. In a badly managed ward, the least bit of extra work puts everything into a muddle, and one accident coming in would make an onlooker think that at least an extensive railway disaster was pouring in its many victims. In such a ward, an order for an ice-poultice presently will reduce the whole kitchen to a state of chaos, and should a dressing be required to be done rather unexpectedly at some inopportune moment, a wild flight will instantly take place of every nurse in the ward, each chasing the other to all parts of the compass, looking for things which should be kept close at hand, while the deserted house-surgeon fumbles helplessly with safety-pins and bandage, outwardly calm, but inwardly wondering what on earth all the fuss is about, and wishing the nurse would be in her place to help get the leg ready for the inspection of his impatient "chief." A new admission will strew the whole ward with clothes, which, picked up from time to time and added to an ever-growing, unsteady pile, meets everyone's eye just in the entrance to the ward kitchen; and the time he takes in getting washed is truly wonderful, while the whole of the linen-cupboard is ransacked to find him a clean shirt, which, when found, is minus buttons and has to be fastened with safety-pins. In a badly managed ward there is always a great deal of talking in the ward kitchen and an enormous amount of running up and down the ward. The bath-room door is generally ajar, revealing forgotten bundles of patients' clothes, old, unclaimed boots, broken vases, dead plants, &c. The nurses are always untidy, late in coming to meals and in going on and off duty. They wear an anxious, worried look, and are tired and depressed. The probationers are constantly called off from their regular work to do something else, so things are never done to time. When the ward is swept the sweepings are hastily gathered into a corner of the kitchen and left there for ever so long, the broom on the top of them. On visiting days patients' friends may be seen sitting on the beds, instead of having chairs given them, and many more visitors than are allowed find their way into the ward. The nurses in such a ward have never done their work, and have very little pleasure in it. And it is all the result of bad management.

It is of little use, though, to go on multiplying instances of bad management, unless we also discuss how it can be avoided. You are all agreed in wishing your own particular hospital to be second to none, and it depends very much on each of you to help bring about that desirable result. In the large London hospitals the ward sister's work is largely one of supervision, but in those of a hundred beds, or less, more of the actual routine duties necessarily devolve upon her. If you then, who are ward sisters in smaller hospitals, sometimes find the work falling rather heavily on the few who know how to do it, in consequence of a small nursing staff, you may take comfort in the thought that it will make you all the better practical nurses, and that your opportunities for seeing and learning your profession are greater for not being crowded out by medical students. Remember that even if your hospital is small, that is no reason why you should be slack, and that good management is just as essential in a ward of ten or twelve beds as if there were thirty or forty.

To have the work well and fairly divided so that in an ordinary day's routine no one gets more than her fair share is one of the prime factors in good management. If you think over the various wards you worked in as probationers, I daresay you can recall many instances in which the lack of this systematic division of labour threw a great deal of extra work on one person.

It is worth while for every ward sister to write out carefully in detail the work of her special ward, and, having written it, to apportion it. A good many nurses do not like the trouble and mental work this entails, and rather than do it they are content to follow in all their predecessor's footsteps, without ever stopping to think if her ways are really the best. It is just by going on blindly like this that mistakes are perpetuated, slovenly ways of doing things countenanced and encouraged, and continued from one generation of probationers to another. Each of you, on taking over a ward from someone else, can at once see some more excellent way of doing something than she did, and also something which you may learn from her. Nobody's methods are so good that they will not admit of improvement, and if you are really in earnest about your work and anxious to do your best for your patients in the very best way, you will, without delay then, review your methods, and see if you cannot bring your ward management up to a higher standard of efficiency than you are doing at present.

*(To be continued.)*

### Echoes from the Outside World.

### AN OPEN LETTER TO A HOSPITAL NURSE.

It is not possible to please everybody, but the new title which it is proposed should be borne by the King has evidently been well considered. For your information I give it in full: "Edward the Seventh, by the Grace of God, of the United Kingdom of Great Britain and Ireland, and of all the British Dominions beyond the seas, King, Defender of the Faith, Emperor of India." The phrase "And of all the British Dominions beyond the seas, King" is, in my humble judgment, very happy. No words could better express the fact, and yet there is a distinct appeal to the imagination which cannot fail to be appreciated, especially in some of the dominions that are indicated. When the necessary Bill has become law, as, of course, it will before the prorogation of Parliament, it may be hoped that the poet will accomplish what the Legislature must necessarily leave undone. The full meaning of the new title should be brought out and popularised in verse by the touch of a master hand.

The Queen's graceful and touching reply to the deputation of the Countess of Aberdeen and the ladies who offered for her Majesty's acceptance a loyal and eloquently worded address, signed by 25,000 of the women of Canada, shows how deeply she appreciates the manifestations of loyalty which originated with the local council of Montreal. The Queen told the deputation that though many assurances of loyalty and devotion had been tendered the King from all parts of the Empire since his accession to the Throne, none would give him greater pleasure than "those which are so kindly expressed in the address presented to me on behalf of the women of Canada;" and she added that the address will always be treasured by his Majesty and herself "not only for the great artistic merit of the volume containing it, but especially for the loving references it makes to her late Majesty Queen Victoria." The address is enshrined in two large and handsomely-bound volumes in heavy yellow leather, the entire surface being ornamented with designs traced in poker work. On the front cover of each are the Royal Arms and the beaver nestling among maple leaves emblazoned in gold and inlaid coloured leather. Some of the names in the album are most interesting, and include those of Lady Thompson, widow of the eminent Canadian statesman who died suddenly at Windsor Castle when he was visiting Queen Victoria; Mrs. Creighton, of Halifax, Nova Scotia, who danced at the ball given in that city in honour of Queen Victoria's wedding; a colony of Danish women in New Brunswick, many French Canadians, and several Indian women and children. The designations of the latter are rather quaint, three of the signatures being Sarah Big Crow, Daring Grasshopper, and Polly Going-to-the-Crees.

Mr. Aston Webb, who is to share with Mr. Brock, R.A., the honour of being associated with the construction of the national monument to Queen Victoria, has quietly, but steadily, risen to the front rank of the profession which he adorns. He is still a comparatively young man, having only just turned fifty, and though his appearance is by no means robust, he is capable of an immense deal of hard work. He restored the church of St. Bartholomew, Smithfield, very successfully, as well as the French Church, Soho, and he was the chosen architect for the completion of the Victoria and Albert Museum at South Kensington, and the Britannia Royal Naval College, Dartmouth, besides having done a great deal of private work. Two years ago he was made an A.R.A., and in all probability as soon as there is a vacancy he will be called into the inner circle at Burlington House. Mr. Webb is passionately fond of his profession and is never happier than when he has his pencil in his hand.

The sketch of Mr. Webb is not yet complete in its details, and it is too comprehensive in its character to be easily understood by the uninitiated; but the principal point in the scheme is the transforming of the space in the front of Buckingham Palace from a large gravelled courtyard as it is at present, into a fit setting for Mr. Brock's great statue of Queen Victoria. The railings are to give way to a stone arcade with side gates, a little suggestive, it is said, of the colonnade of St. Peter, Rome. In this manner the great gates in the centre through which so many of us have often watched the late Queen emerge upon State occasions, or when she was staying in town, will be eliminated. From where the sentry boxes now stand the arcade will curve inwards, and there the necessary space will be gained for Mr. Brock's memorial. The roadway from Constitution Hill will be given a much wider curve, and so will the continuation of the Mall leading to Buckingham Palace Road. In fact, the road is to be thrown back as far as the lake, and gardens made in place of the road, which will be public, gay with flowers and green with grass, and perhaps beautified by two small lakes. The scheme as at present designed is to cost the £130,000 already subscribed. Its elaboration will depend entirely upon the amount of money which the committee may eventually have to spend.

As to the statue itself, it will be raised about 8 feet from the ground, and broad, easy flights of steps will probably form the approaches from the front and back. In the centre will rise a pedestal which will attain about 68 feet in height. On the left and the right will be two basons in the shape of a new moon, a fountain breaking the line of the wall. In the front of the monument will be seated the figure of Queen Victoria herself in regal robes. On her right hand a group of statuary representing Truth is contemplated; on the left another emblematical of Justice. Other figures are to typify Maternity, Courage, and Constancy; and surmounting all Victory is to appear. The water arches will have upon them reclining figures to illustrate the Army on one side, the Navy on the other. The whole will, of course, be of immense proportions, and will need to be seen to be realised, as figures and descriptions convey so little to the ordinary mortal.

Nurses whose work lies among the miners in the North of England, and who know something of the influence which the Bishop of Durham exerted among the toilers in the bowels of the earth, will deeply regret the death of Dr. Westcott, which was announced on Monday. Fitly enough, the last sermon which the Bishop delivered in Durham Cathedral was on Saturday week in the presence of a crowded congregation of miners, to whom he addressed some final words of counsel; for he did not, in any case, intend to remain in charge of the see. He has, however, been removed before he could resign, and the northern diocese has thus lost a prelate who, though at once a ripe scholar, a profound theologian, a brilliant critic, and a capable administrator, will, perhaps, be most lovingly remembered as a Peacemaker. His labours to improve the relations between the coalowners and their men, and the tact and the Episcopal gentleness which he manifested during the time of the great strike in 1892, will not soon be forgotten. Moreover, his efforts were attended with complete success, for it was mainly through his instrumentality that the costly struggle was brought to an end. Lord Salisbury will find it no easy matter to choose a suitable successor to two such bishops as Lightfoot and Westcott.

Everywhere on the Continent the position of women is becoming more important except in Turkey, where, owing to a recent decree of the Sultan, affairs are in a retrograde condition. It is not long since the first Turkish girl graduate won for herself distinction, which fact has apparently alarmed the powers that be, for new measures of a stringent character have been ordered, which forbid any Turkish children to attend foreign schools, or the heads of families to employ foreign governesses or teachers. This means that education, which of late had become quite fashionable in the better-class families, will have to cease.

### Everybody's Opinion.

[Correspondence on all subjects is invited, but we cannot in any way be responsible for the opinions expressed by our correspondents. No communication can be entertained if the name and address of the correspondent is not given, as a guarantee of good faith but not necessarily for publication. or unless one side of the paper only is written on.]

### SELECTION OF PLAGUE NURSES FOR DUTY IN SOUTH AFRICA.

"Another Plague Nurse," attached to the Plague Camp at Uilvengo, under date of June 8, writes: A copy of The Hospital Nursing Mirror, dated June 1, reached us by the last mail, and it was a great surprise to the nurses to find therein a letter written by one of the twenty. What was the writer's motive? "Jealousy?" Was she herself such a pattern of discretion on board ship? Let her ask herself this question: "Before I accuse my fellow workers of anything, am I above reproach?" Again, the majority were never consulted on the subject, therefore they could not be said to agree with the correspondent. Besides, surely our matrons at home have not had these nurses under their supervision for three years or more without being able to judge of the individual character of each nurse. Ought not the correspondent to take herself to task for daring to question the duties of a matron? Is she the only one out of the twenty who is going to do credit and uphold the standard of the profession?

### PRIVATE NURSES' FEES.

"Bede" writes: Is "A. B.'s" attack on private nurses' fees a pure effort to raise the salaries of institute nurses? If so, why not at once declare that they are underpaid instead of suggesting that private nurses are overpaid? If it is a desire to serve the public, surely that body (the sick members are happily in the minority) is best served by honourably supporting those amongst women workers who dare to acknowledge their individual responsibilities, risking the direct attack of censure or praise, unshadowed by the prestige or chaperoning influence of an institute. The chaperoning system is good in its place, but it is a means, not an end; used it is discipline, abused it is bondage. The individual worker must always command a higher price than one who shelters in a community for many practical reasons. And it is inevitable if a man or woman chooses to resign an iota of his individual fight for existence, either from dread of the risks with its anxieties, or from mental laziness, that he becomes the loser, not only in purse, but in character.

### NURSES WHO SHOULD BE WEEDED OUT OF THE PROFESSION.

"The Matron" of the Nurses' Co-operation and Home, 53 Fylde Road, Preston, writes: Can anything be done to prevent nurses from remaining in the profession, who so far forget themselves as to disgrace both their uniform and sex by getting drunk on duty? We all know what it would mean for a soldier; then why should a woman who has people's lives in her charge escape? Drink is not the only cause that should cut short a nurse's career, as I have good reasons to know. I find that references are nothing to depend on when engaging a nurse; and as for testimonials they are often not worth the paper on which they are written. I wonder if it ever strikes people who write such references the harm they are doing the general public, the medical men, the really good, reliable nurse (as I believe the majority are), and the matron who is so unfortunate as to get such women on her staff. The most annoying part of the matter to me is this: the matron dismisses the nurse, who promptly looks out for another post with the same credentials she has fooled other matrons with, and thus ends the matter for the nurse, who takes great care not to let her future address be known if she can help it. On the other hand, the matron has to lose the fees from the case when the nurse has misconducted herself, and run the risk of her home being cried down by the patient's relations and friends, who if they are unreasonable people, blame the matron for the nurse's shortcomings. Then rumours get afloat to the effect that the nurses from that home are no good. This is a most serious thing to any matron anxious to have a home with a good name, and to all other nurses who are doing their level best in the profession. I would suggest that a public register be kept where the names of nurses may be entered, with causes of dismissal from hospital or home, and perhaps funds could be raised for a home for the dipsomaniac, who is nine times out of ten a very clever woman, and is as much to be pitied as blamed. How or why they have become dipsomaniacs is best known to themselves, but I do know that often the public are to blame for not providing proper meals for the nurses engaged at serious cases. The patient's friends forget that the nurse is only a woman, and that she is not trained to do without food or sleep. This home up to the present has only had thoroughly trained nurses on the staff engaged at a salary of £35 per annum with laundry, with as comfortable a home-life as I can give them: they are allowed to bring their friends to the home, and it is no uncommon thing for me to relieve a nurse engaged at a serious case to enable her to get a little sleep when the patient cannot afford two nurses. A nurse may also have a bed from the home for her own use when the sleeping accommodation is limited. I thought that by doing my part towards the nurses they would do theirs to me. Some nurses who have been dismissed from here have described my home as a hovel, &c. (I trust this letter will come under their notice.) This town is not very well supplied with district nurses, and we are so situated as to be able to be of great use to our poorer neighbours; but some nurses have refused to do little kindnesses for such people, although in the home doing nothing. Others have gone when requested, but at the same time they considered it degrading, and said (behind my back of course) that I was no lady to do it myself or to ask them to do it. Now are such women fit to be nurses? And can you wonder that some of the general public have such a rooted dislike to the private nurse, and that we are so often regarded with suspicion when we enter a house of sickness by the patient's friends and servants, if there are any, the latter making up her mind to plenty of extra work, and vowing to herself not to run up and down after that nurse. Yet how soon is the feeling dispelled with a little tact from a good nurse, who will always accommodate herself to any class of people or surroundings. I have generally found that the nurse who wants weeding out of the nursing profession is extravagant, lazy, and untidy to a degree, and when she does go out to a case, instead of falling in with people's ways and means, and trying to make things run as smoothly as she can for the people who more often than not are trying to make her as comfortable as they know how, she simply sits down and makes herself as objectionable as possible by giving herself the airs of a duchess, talking about her imaginary wealthy relations and various other idiotic topics, such as being able to leave off nursing any day, as there is no actual necessity for her to do such laborious work. She thinks that by emptying slops, &c.—which are essentially nurses' duties—she is doing something very much beneath her, although she is not above gossiping with the servants, retailing the patient's affairs, and will even go so far as to question the medical attendant's treatment of the case. I give my address; for this home, small as it is, will bear the closest scrutiny; and I know that my in-patients and nurses will testify to the truth of this letter. A great deal is expected from the private nurse by the public; so let the public remember there is a great deal due to the nurse, who too often works 16 and 18 hours out of the 24 for the welfare of her patient, never thinking of herself or what it will mean to her if she does it once too often. I trust that this will not take up too much space in the nurse's invaluable paper.

### The Nurses' Bookshelf.

Bacteriology and Surgical Technique for Nurses. By Emily M. A. Stoney, Superintendent of the Training School for Nurses, St. Anthony's Hospital, Rock Island. Ill. (London: W. B. Saunders and Co. 1900. Price 5s. net.)

Take it altogether this is a good and useful book. There is much in it that the ordinary nurse certainly does not want to know, and a good deal the attempt to understand which would probably lead to confusion in her mind; nevertheless to a nurse of intelligence who wishes as far as may be to know something about the processes which are constantly going on under her eyes, and to see reasons for the things she has to do, this book will be found a very useful guide and companion. The chapter on bacteriology gives a good general sketch of the rôle of the microbe in the causation of disease, and forms a useful introduction to that on antiseptics and disinfectants. The larger portion of the book, however, is devoted to surgical "technic," as it is called in the pages, notwithstanding that the word "technique" is used on the cover. We think that the term "surgical technic," as applied to the work of the nurse in preparing for and assisting in operations, is somewhat of a misnomer; but there is no doubt that this section of the work before us is very thorough and complete, and that if a nurse will carefully read it and will compare the detailed directions given in it with what she actually does in her daily routine she will find much to think about. There are chapters on the care of the operating room and of instruments. Lists of the instruments required for various classes of operation are given; there is a chapter on anæsthetics, dealing chiefly with American methods, ether being described as given by the open method; a very good chapter on surgical dressings, saline solution, irrigation, etc.; and full details in regard to the preparation of the patient and the operating-room. All of these are very carefully done, and at the end of the book there is a useful chapter on the arrangements required when a post-mortem examination has to be made in a private house. All nurses will of course know that the antiseptic routine adopted at different hospitals and by different surgeons varies a good deal, notwithstanding that its aim and object is always the same, namely, the protection of the patient from septic infection; and they will therefore remember that the exact directions laid down by the author of this book permit of many modifications by different surgeons. But the directions given are good, and wherever a nurse is thrown on her own resources, and has to act without instruction, she will be safe in doing as the authoress here tells her, for her standard is a high one.

Thirty-one Twenty-four Hour Charts for Private Nurses. By Florence White. (London: James Townsend and Son, Stonecutter Street. Exeter: Little Queen Street. Price 1s. 1d.)

This arrangement for the plain statement of the patient's condition every 24 hours will certainly be found very useful both to the trained private nurse and to the amateur, for it will simplify matters for them and enable the doctor to see at a glance his patient's state, or at least, let us say, his state as it appears to his attendant, without having to wade through wordy statements. In the hands of the amateur, it would perhaps be better to leave the lines for pulse and respiration alone and avoid worrying the invalid for that "which profiteth not." The doctor will attend to these matters when he comes, and if he considers it necessary that they should be noted between his visits, presumably he would consider the patient too ill to be in unskilled hands.

### Appointments.

Croydon Union Infirmary.—Miss Isabel F. H. Buchanan has been appointed superintendent night nurse. She was trained at Crumpsall Infirmary, where she has since been ward sister.

Dundee Parochial Hospital.—Miss Jessie Maclaren and Miss Jane Milne have been appointed charge nurses. Miss Maclaren was trained at Burton Infirmary, and has since been nurse at Dunfermline Poorhouse Hospital. Miss Milne was trained at Barnhill Hospital, Glasgow, where she has since been staff nurse.

Hospital for Soldiers' Wives and Children, Woolwich.—Miss A. E. Ashman has been appointed sister. She was trained at St. Pancras Infirmary, and worked for two years at Miss Morgan's Nursing Institution at Shrewsbury. She holds the L.O.S. certificate.

Longford County Infirmary.—Mrs. McDonnell has been appointed matron. She was trained at the Rotunda Hospital, Dublin, and has since been attached to the Trained Nurses' Home at Birkdale.

Park Hospital, Hither Green, Lewisham. — Miss Mildred Annie Fletcher has been appointed charge nurse. She was trained at St. Pancras Infirmary.

Queen Victoria's Hospital, Las Palmas.—Miss Kate V. Mallinson has been appointed nurse. She was trained at Chorlton Hospital, Manchester.

Reigate Isolation Hospital.—Miss Amy M. Hore has been appointed matron. She was trained at Addenbrooke's Hospital, Cambridge, and the London Fever Hospital. She has since been night superintendent at Addenbrooke's Hospital, and has had charge of the diphtheria ward at Park Fever Hospital.

Royal Infirmary, Preston.—Miss L. C. Gibbon has been appointed assistant matron. She was trained at King's College Hospital, London, and Salop Infirmary, Shrewsbury. She was subsequently night superintendent at South Devon and East Cornwall Hospital, Plymouth.

Ryde County Hospital.—Miss Emily Hudson has been appointed sister. She was trained at Poplar Hospital for Accidents, where she has taken holiday duty.

St. Luke's House, Lawn Road, Hampstead.—Miss Amy F. Lang has been appointed matron. She was trained at St. Bartholomew's Hospital, and has since been private nurse, superintendent of nursing at the Greek Hospital, Alexandria; temporary matron at the Hospital for Epilepsy and Paralysis, Regent's Park; and superintendent of nursing at the Hampstead Infirmary.

Tunbridge Wells General Hospital.—Miss E. M. North has been appointed sister of the women's wards. She was trained at the Hospital for Diseases of the Heart, Soho Square, the Great Northern Central Hospital, and Queen Charlotte's Hospital, London. She holds the L.O.S. and Q.C.H. certificates. Miss North has recently held the post of night sister at the Croydon General Hospital.

Walsall Workhouse Infirmary. — Miss Florence Baker has been appointed charge nurse. She was trained at Stanport Union Infirmary, and has since been charge nurse at Bury Union Infirmary, Lancashire.

Warwick Infectious Hospital.—Miss Gertrude Digwood has been appointed matron. She was trained at Warneford Hospital, Leamington, and has since been matron at the Cottage Hospital, Pershore. She was for six months with the Field Hospital Army Medical Staff in South Africa.

West Bromwich District Hospital. — Miss E. W. Stephens has been appointed sister. She was trained at the Royal Women and Children's Hospital, Bristol, and the Royal Infirmary, Bristol. She has since been engaged in private nursing at the Sussex County Hospital, Brighton. Miss Stephens holds the L.O.S. certificate.

### For Reading to the Sick.

"WAIT THOU IN HOPE."

WAIT on the Lord for what He hath to give,
O restless heart;
He knows the sorrows that beset thy way,
He knows thy fretful weariness to-day,
O fainting heart!

When thou hast stilled thyself to rest in Him,
O throbbing heart;
When thou hast learned to love Him first and chief,
To love Him even better for thy grief,
O weeping heart!

Then will He grant thee all thine own desire,
O longing heart;
Sunlight of joy may even here be given
If so He will—if not, sunrise in heaven,
O waiting heart!

*M. E. Townsend.*

. . . Jesus said
His followers should have a hundred-fold
Of earth's most precious things, with suffering.
In all the labourings of a weary spirit,
I have been blessed with gleams of glorious things.
The sights and sounds of nature touch my soul,
No more look in from far. I never see
Such radiant, filmy clouds, gathered about
A gently opening eye into the blue,
But swells my heart, and bends my sinking knee,
Bowing in prayer. The setting sun, before,
Signed only that the hour for prayer was come,
Where now it moves my inmost soul to pray.

*G. Macdonald.*

"My soul, hope thou in God, for I shall yet praise Him who is the health of my countenance and my God." This hope was *in God.* The mistake we make is to look for a source of comfort in ourselves; self-contemplation; instead of gazing upon God. In other words we look for comfort precisely where comfort can never be. For, first, it is impossible to derive consolation from our own feelings, because of their mutability; to-day we are well, and our spiritual experience, partaking of these circumstances, is bright; but to-morrow some outward circumstances change—the sun does not shine —and we are gloomy, low, and sad. Then if our hopes were unreasonably elevated they will now be unreasonably depressed, and so our experience ebbs and flows, like the sea, that emblem of instability. Next, it is impossible to get comfort from our own acts; for though acts are the test of character, yet in a low state no man can judge justly of his own acts.

God is not affected by our mutability; our changes do not alter Him. When we are restless He remains serene and calm; when we are low, selfish, or dispirited, He is still the unalterable I AM—the same yesterday, to-day, and for ever, in whom is no variableness, neither shadow of turning. What God is in Himself, not what we may chance to feel Him in this or that moment to be, that is our hope. "My soul, hope thou *in God.*"—*Fosberry.*

### Notes and Queries.

**The Editor is always willing to answer in this column, without any fee, all reasonable questions, as soon as possible.**

**But the following rules must be carefully observed:—**

1. **Every communication must be accompanied by the name and address of the writer.**
2. **The question must always bear upon nursing, directly or indirectly.**

**If an answer is required by letter a fee of half-a-crown must be enclosed with the note containing the inquiry.**

*South Africa.*

(174) I should like to go out as nurse to South Africa; can give the very best of references. I had a chance of going out at the beginning of the war, but to settle in the country so as to be well known amongst the doctors is my object, and having three brothers at the war they can recommend me to several doctors they know. May I ask you to kindly send me particulars where to apply, etc.?—*Hospital Nurse.*

One of the several doctors known to your three brothers would probably be able to tell you where there is an opening.

*Phthisis.*

(175) A poor man, in whom I am interested, desires that his little daughter, believed to be in consumption, should be placed under the open-air treatment. Is there any suitable home? The father can afford to pay a trifle. *J. B.*

The North London Hospital for Consumption and Diseases of the Chest, Mount Vernon, Hampstead, N.W., meets the requirements of the case.

*Young Probationer.*

(176) Is there any general hospital where a young lady of 20 could begin training without premium?—*V. M. J.*

I am nearly 18 years of age; are there any hospitals in London where probationers of that age are received?—*Jeannie.*

I am nearly 18, and have answered several advertisements in THE HOSPITAL, but find I am too young. Would I be able to get into a children's hospital?—*R. B.*

Young probationers cannot stand the strain of training in a general hospital, hence the refusal of good schools to accept them. Study domestic duties until you are of suitable age.

*Sanitary Inspector.*

(177) 1. How can I be qualified as Lady Sanitary Inspector? 2. What qualifications must I have to enable me to take a post under the London School Board for physically defective children?—*J. B.*

1. You must satisfy the examiners of the Sanitary Inspectors' Examination Board, 1 Adelaide Buildings, London Bridge. E.C., or of the Sanitary Institute, Margaret Street, W. Apply to the Secretaries for Syllabus. (2) Write up to the Secretary, London School Board, Victoria Embankment, W.C., for particulars.

*Up-Country Nursing Association.*

(178) Will you kindly tell me where I can get the rules and regulations of the Up-Country Nursing Association in India?—*Mrs. C. and J. F.*

Major-General G. Bonus, The Cedars, Strawberry Hill, is Hon. Secretary.

*Second-hand Chair.*

(179) Can anyone help me to get, second-hand, a self-propelling wheel or bath chair? It is for a poor half-paralysed villager who is unable to get out. If he had a wheel chair his mother could move him single-handed. The chair, therefore, would be the greatest boon, and I think the necessary money for a second-hand chair could be raised amongst the parishioners.—*E. M. B.*

Have you tried advertisement or the "Bazaar or Exchange and Mart"?

*Home for Servant.*

(180) I should be very grateful if you could recommend a comfortable home where a valuable and good servant would feel happy. She is slightly afflicted with creeping paralysis, and feels her inability to work keenly. She could afford to pay 18s. or 20s. a week.—*Nurse Martin.*

One of the following homes might be found suitable:—St. Peter's Memorial Home, Maybury Hill, Woking; Woodside Home, Whetstone, N.; St. Marylebone Home for Incurables, 61 Weymouth Street, Portland Place, W.; St. Cyprian's Home, 31 The Grove, Hammersmith; or 10 Little Park Street, Dorset Square.

*Holiday Home.*

(181) Can you give me the name of a holiday home, on the West Coast preferred, where two children, who are out of sorts, could be received for a month or a fortnight for a small sum weekly?—*E. D. K.*

There are convalescent homes for women and children at Weston-super-Mare: The Children's Convalescent Home, Clifton Road, 8s. a week; and the Royal West of England Sanatorium, with subscriber's letter 5s. a week. Also at Clevedon: The Convalescent Home, Belmont, Highdale Road, with subscriber's recommendation 5s. a week. At Lymington: The Cottage Convalescent Home, 7s. 6d. a week. Or you will find many advertisements of the kind you want in the daily papers.

**Standard Books of Reference.**

"The Nursing Profession: How and Where to Train." 2s. net; post free 2s. 4d.

"Burdett's Official Nursing Directory." 3s. net.; post free, 3s. 4d.

"Burdett's Hospitals and Charities." 5s.

"The Nurses' Dictionary of Medical Terms." 2s.

"Burdett's Series of Nursing Text-Books." 1s. each.

"A Handbook for Nurses." (Illustrated.) 5s.

"Nursing: Its Theory and Practice." New Edition. 3s. 6d.

"Helps in Sickness and to Health." Fifteenth Thousand. 5s.

"The Physiological Feeding of Infants." 1s.

"The Physiological Nursery Chart." 1s.; post free, 1s. 3d.

"Hospital Expenditure: The Commissariat." 2s. 6d.

All these are published by THE SCIENTIFIC PRESS, Ltd., and may be obtained through any bookseller or direct from the publishers, 28 and 29 Southampton Street, London, W.C.

### Travel Notes.

By Our Travelling Correspondent.

### LXXVII.—TO THE CONTINENT *VIA* ANTWERP.

It is the usual thing to visit Belgium and the Ardennes, passing by Antwerp, the port of entrance, as if it were a place beneath notice, in our hurry to reach our bourne further on in old Flanders or the green and luscious valleys of the Ardennes. I plead now that you should make a short stay in the ancient city, once so powerful and so rich, and still full of historic memories of the glories and the sufferings of the past. Get from your library Motley's "Rise of the Dutch Republic"; it is a fascinating book, and reads like a long and exciting novel.

### Arriving at Antwerp.

There are several routes and all are cheap: *via* Harwich, second-class return is £1 4s., and there is a cheaper way from the Tower Bridge; but those boats only run on certain days. There is also a service of boats direct from Hull, and another from Leith. From Hull, second return, £1 5s.; and from Leith, £2; also from Newcastle, £1 10s. This is very convenient for those living in the north, sparing them the expensive journey south by rail.

### The Approach to Antwerp.

I must candidly say that it is not prepossessing; the country lies so low and so flat that steaming slowly up the crowded Schelde, one positively looks down upon the small, uninteresting villages and (as one nears Antwerp itself) upon the desolate-looking suburbs now extending far beyond the lines of the fortifications. My first visit was in 1882, and I took the cheap line from Tower Bridge—fares 16s. first-class single, and 11s. second-class single. It is a long voyage that way, and therefore unsuited to those who have but little time at their disposal; but the boats are excellent, and the journey interesting, because of the river life from Tower Bridge to Southend.

Luggage is examined on board, which is a saving of time and trouble. The last time I went through the ordeal, an elderly spinster, whose bag was being rummaged, remonstrated in loud tones: "My name is B——, and I am going to Brussels, where I am greatly respected and beloved, and anyone will tell you I am perfectly honest." The stolid Flemings returned no answer to this impassioned appeal, but continued their search with renewed vigour.

### The Sights of Antwerp.

There are several very cheap and good hotels in Antwerp where you may spend two or three nights with little expense and see the pictures and the architecture of the old city. Alas! the odious hand of the municipal improver has lain heavy on Antwerp. For instance, the Steen prison, one of the most interesting buildings in the town, still exists, but all the surrounding streets and old houses have been demolished, leaving it standing naked and alone like a gigantic baby-house. A few years ago there was a fine old Calvary close by, at which prisoners knelt and prayed, often for the last time before they entered the gloomy Steen and looked their last upon the blessed light of day.

There are numerous torture chambers below, all designed with much ingenuity to give the maximum of suffering. There is the room for the torture of the drop, where the prisoner, bound immovably, was placed beneath a tap from which a single drop exuded every two or three seconds on the victim's head. Another tiny room constructed without a window but a large fireplace, was admirably contrived; a large fire was made up, the prisoner was put into the room (little bigger than a cupboard), where in a short time, maddened by the heat, he ran from side to side. Then the watchful warder suddenly opened the door, and the doomed man rushed out and fell through a trap door into a canal (underground) which swept the body out to the sea.

### The Cathedral and Other Churches.

On Sundays and Thursdays from 8 to 12 you can see Rubens' great pictures free; at other times a charge of 1frc. per head is made. The splendour of the building is beyond praise. It is so unusual to see six aisles, and the effect is very grand. One regrets the absence of coloured glass; had it the windows of the Dom in Cologne what a building it would be! There is fine music at High Mass on Sundays. To hear this and see the pictures it is well, if possible, to arrive at Antwerp on Friday, spending Saturday in other sights and leaving Sunday for the cathedral and the music, also free on Sundays. In one of the north aisles is a very quaint picture by Teniers. I never omit to visit it when in Antwerp. It illustrates the seven acts of mercy: one man, very dumpy, is distributing loaves of enormous proportions to a voracious crowd, another party of the godly is visiting the sick, who, very much in evidence, are up in "a two pair back" with the wall of the house taken out, that they may be the better in view. Then comes clothing the naked, who are coolly walking about the streets of Antwerp in a state of nature, apparently in no way embarrassed by their unconventional condition. One man is peering in a shortsighted manner through a grating at some prisoners, very large in proportion to their cage; but I think the gem of the series is succouring, the homeless—several patched and tattered travellers are prancing gaily into a mediæval house at the invitation of a mail-clad warrior, and in the foreground, like followers of Messrs. Cook and Gaze, numerous homeless repose, two of them hugging a veritable hold all!! The Dominican Church of St. Paul comes next in interest; here the carving of the confessionals is wonderful, and outside the Church is an extraordinary Calvary; it is impossible to call it beautiful in any sense, but it is singular. Imagine a species of suburban grotto of large dimensions thickly "planted with saints," as the local guide describes it; nothing can be more lacking in taste, but it is interesting to note that the horrors of rock work and grottoes are by no means modern atrocities.

In the church of St. Andrew there is a wonderful pulpit representing the Disciples in a boat, and our Lord walking on the sea. The figures are life-size, and their action excellent.

In St. Jacques Rubens is buried in what is called the Rubens Chapel, under one of his own fine altar-pieces.

He was born in a house in the Place de Meir; this house, much altered, still stands, and its owner kindly allows enthusiastic pilgrims to enter the garden and the summer-house, which Rubens used as a studio.

---

### TRAVEL NOTES AND QUERIES.

Rules in Regard to Correspondence for this Section.—All questioners must use a pseudonym for publication, but the communication must also bear the writer's own name and address as well, which will be regarded as confidential. All such communications to be addressed "Travel Editor, 'Nursing Mirror,' 28 Southampton Street, Strand." No charge will be made for inserting and answering questions in the inquiry column, and all will be answered in rotation as space permits. If an answer by letter is required, a stamped and addressed envelope must be enclosed, together with 2s. 6d., which fee will be devoted to the objects of the "Hospital" Convalescent Fund. Any inquiries reaching the office after Monday cannot be answered in "The Mirror" of the current week.

Lausanne for Three Months (Zero).—There is plenty of accommodation at moderate terms in and about Lausanne, but opinions differ as to what is moderate. Tell me what you are prepared to pay and I will advise you. July, August, and September are the three dearest months in Switzerland.

Lower Brittany (Asphodel).—The sort of place you want is Pornichet, I think. It is good for children, being safe; indeed, I am told there are rather too many of those small gentry, but I have only been there in May and June, which is not holiday time. It is not far from Saint Nazaire. The best route is viâ St. Malo.

Knokke for August (Pebble).—It is very reasonable, but August is a dearer monrh than June; still even then I think it will be within your means. You will find a full account of Knokke in the Nursing Mirror of September 1st, 1900. There is a good deal to be seen, and trains and trams are convenient, taking you easily into Holland. By going there you could join in the gaieties of Ostend without paying the price of the dear hotels.

Verona for a Week (Malaga).—I do not think you can do better than the Colomba d'Oro. It is reasonable and clean. Verona is not very luxurious in the matter of hotels. It is a bad place for mosquitoes; arrange some sort of net for yourself; they do not supply them as at Venice, and yet it is quite as necessary.

